# Accidents Caused by a Very Small Dose of Santonine Given to a Child

**Published:** 1852-03

**Authors:** 


*Accidents caused by a very Small Dose of Santonine given to a Child.*—Santonine, being a tasteless vermifuge, is easily given to children, consequently its employment becomes daily more and more frequent ; we therefore think it useful to expose the accidents which may follow the use of this medicine, when given in too large a dose. We refer to a case given in the Bulletin de Therapeutique, by Dr. Spengler (d'Herborn). The patient, a child of four years old, who had been suffering for several months from intestinal worms, had taken at different times, and with success, a dose of a grain and a half. One day they gave him three grains in two doses ; after the first dose he became troubled with pains in the epigastrium, colic, and vomiting. He had frequent stools, in which were found a number of ascarides. Notwithstanding these numerous evacuations, the bad symptoms continued to increase ; his body became cold, his face livid, his eyes had a blue circle round them, a cold sweat broke out, his respiration became embarrassed, and his extremities convulsed. Besides these symptoms, M. Spengler mentions that there were dilatation of the pupils and great pain in the abdomen (not, however, increased by pressure). He prescribed milk in abundance, and after several evacuations, the potion of Riviere in an oily emulsion. The little patient was placed in a very warm bed ; during the night he was much disturbed ; the following day he took some doses of calomel, after which several worms were evacuated, and from that time he became convalescent. We have related this fact as a caution against the accidents which may result from the use of santonine, although the severity of the symptoms and the smallness of the dose may make us doubt whether the santonine was pure, or whether some other cause might not have produced the terrible results attributed to it.—*Journal de Pharmacie et Chimie.*

